# The 'off-hour' effect in trauma care: a possible quality indicator with appealing characteristics

**DOI:** 10.1186/1757-7241-19-33

**Published:** 2011-06-09

**Authors:** Stefano Di Bartolomeo

**Affiliations:** 1 Anaesthesia and ICU, University-Hospital, Udine, Italy/Regional Health Agency of Emilia-Romagna, Bologna, Italy

## Abstract

A recent paper has drawn attention to the paucity of widely accepted quality indicators for trauma care. At the same time, several studies have measured whether mortality of trauma patients changes between normal working time and other parts of the day/week, i.e. the so-called 'off-hour' or 'weekend' effect. This measure has the characteristics to become an accepted quality indicator because it combines the advantages of both outcome and process indicators. As an outcome indicator it would not need validation, a procedure particularly difficult in trauma care where gathering scientific evidence is more difficult than in other disciplines. As a process indicator it would provide indications about where to intervene to improve quality.

## Introduction

Although the importance of quality indicators (QI) is undisputed, the debate concerning their validity is incessant. A recent systematic review [1] concluded that 'there is not a common set of clearly defined, evidence-based and broadly accepted QIs for evaluating the quality of trauma care.'

A recent study [2] compared the mortality of trauma patients admitted inside and outside normal working hours in a North-American trauma system. Evenings, nights, and weekends were intended as non-working hours - also referred as 'after' or 'off' hours, as opposed to 'office' or 'business' hours. The study found no difference, however previous studies that investigated the so-called 'weekend' or 'off-hour' effect in various diseases yielded inconsistent results; sometimes such a difference was found [3-8,15] and sometimes not [9-15].

This commentary discusses why the evaluation of the 'off-hour' effect can also be considered a QI. Furthermore, it examines the theoretical characteristics of such a QI, with a special emphasis on its potential to overcome the usual obstacles for QIs in trauma care.

## Discussion

### Quality indicators and scientific evidence

QIs aim at measuring quality. The common definition of quality by the United States Institute of Medicine is 'the degree to which health services for individuals and populations increase the likelihood of desired health outcomes and are consistent with current professional knowledge [16].' Thus, any QI should be related to a certain level of the desired health outcomes. Any person is allowed to consider an outcome as 'desired' and devise the consequent QIs. Nevertheless, there is little doubt that health-care quality ultimately aims at influencing mortality and/or morbidity. Indeed, the above mentioned two outcomes are most used QIs themselves, under the category of 'outcome indicators'' of the classic classification by Donabedian [17]. However, it has been identified that the effects of quality on mortality may be difficult to measure because of a low signal-to-noise ratio [18]. It has been suggested to measure the processes of care (by the so called 'process indicators') instead of the outcomes to overcome the above-mentioned problem [18]. However, in order to improve the quality, the processes measured by such QIs should 'increase the likelihood of desired health outcomes.' Therefore, 'out through the door, in through the window' is the link with survival [19]. The link is usually provided by research and represents the evidence underpinning the QI itself. For instance, first a good level of evidence (i.e. a survival benefit) was established by scientific research for administering beta-blockers in the emergency room to patients with myocardial infarction. Subsequently, a QI measuring the actual adherence to this practice was widely adopted [20,21]. Further attempts to validate this QI proving its link with the outcome (i.e. comparing patient survival in the hospitals with high adherence vs. hospitals with low adherence) may be desirable, but not indispensable.

### Quality indicators in Trauma Care

Trauma care, as compared to other branches of medicine, suffers from a paucity of evidence, as a result of underfunding of research [22]. In addition, special difficulties in collecting the information due to some characteristics of trauma care itself, such as multidisciplinarity, logistic complexity, and emergency also result in insufficient evidence. Therefore, a majority of the processes of care are not supported by evidence. Subsequently, the respective QIs are also not supported. The ensuing attempts to validate these QIs (i.e. the assessment of their relationship with the patients' outcomes, usually mortality) are not substantially different from the scientific research into the processes themselves, and are hindered by the same difficulties. Thus, such attempts are often unsuccessful [1,23-25]. For example, it is reasonable for an Emergency Medical System to evaluate its quality through the rate of prehospital intubation of head-injured patients with GCS <9. However, if a researcher sought to validate this indicator against survival (the 'golden' outcome), he/she would face the same uncertainties faced by intubation itself [26].

Hence, it is not by chance that the most used and accepted QI in trauma care is a straightforward outcome measure, i.e. the benchmarked risk-adjusted mortality. The main advantage of the above mentioned QI is that it does not need validation. However, the main disadvantage is that it does not refer to specific processes of care. As a consequence, the quality-makers remain in search of valid process indicators at the time of identifying and targeting the causes of mortality differences.

### The 'off-hour' effect as a quality indicator

Mortality in 'after time' versus 'business time' expresses whether the quality of care is the same during the different time periods being compared. This analysis is meaningful and of practical interest as everybody is aware of the possible deficiencies in trauma care during after-hours. Such deficiencies are caused by the differential availability of staff, facilities, resources and procedures, by fatigue or sleepiness of the personnel and by increased logistic difficulties in pre-hospital rescue (e.g. flight restrictions for helicopters at night).

Similar to the benchmarked risk-adjusted mortality, the investigation of the 'off-hour' effect would enjoy the important benefit of being an outcome indicator. Therefore, this indicator would not require validation against the outcome. At the same time though, differently from the benchmarked adjusted mortality, it does not measure the quality of care on the whole, but just a portion of it. Therefore, it could act as a process indicator as well, and help identify the processes that should be targeted to improve the quality of care. For example, this QI might drive interventions to increase the staffing of hospitals during weekends or launch a night flight HEMS program. Moreover, the re-calculation of the QI at a later time could assess the efficacy of the above-mentioned interventions. On the other hand, the absence of the 'off-hour' effect could be a sort of a quality mark for hospitals or systems whose specific processes of care could then become models for others to copy.

Another advantage is that this QI can be calculated at the local level (trauma center, trauma system or geographical region) without complex benchmarking against data from other settings, a procedure that may be biased if the data are inhomogeneous. The evaluation of the 'off-hour' effect is an internal comparison, as the compared groups come from the same setting. Thus, unaccounted differences (e.g. systematic between-hospital differences in severity score assignment) are less probable. Conceptually, it resembles the difference that occurs between the case-control and case-crossover study-design [27]. In the former design, cases and controls are different subjects, while in the latter design cases and controls are the same subjects, though observed in different times. Consequently, some sources of potential confounding, i.e. those related to the fixed characteristics of the unit of analysis do not change within the matched pairs and are controlled for by the design.

However, it is necessary to exercise some caution and understanding. All the factors influencing survival at the patient level (age, mechanism of injury, injury severity etc.) should be carefully accounted and adjusted. This is because systematic differences may still occur. For instance, patients admitted in the off hours are known to be younger, [2] plausibly because the young tend to go out at night. In addition, penetrating trauma occurs more often, [2] probably as more violence transpires at night. Finally, injury severity may be worse because traffic accidents are also more severe at night [28]. For all the above-mentioned reasons, a crude, unadjusted comparison of mortality would not be reasonable. Thus, a risk-adjusted model would be required for a proper application of this QI.

Another important caveat is that the aspect of quality measured by this indicator is relative, and not absolute. In other words, the absence of the 'off-hour' effect is always recommendable, but not sufficient. Even though it is uncommon, a system with the same mortality in working and off hours could still have an elevated overall mortality, which is disturbing. Therefore, this QI should not be considered as an alternative to the benchmarked risk-adjusted mortality, but only as complementary.

This QI would retain its meaningfulness when applied at any level (e.g. one or more hospitals, one trauma system or one geographical area). However, it could capture the full picture of possible differences between parts of the day/week only if all the possible hospitals where a patient could be brought were included. The processes of care that bring patients from the trauma scene to the definitive hospital are crucial [29]. Further, these processes are also likely to be affected by the time of the day. The processes of care would be fully mirrored if the indicator were applied at a population-level, i.e. trauma system or geographical area. Suppose, for instance only some hospitals within a system (usually the referrals centers) are considered and the patients transferred from another facility are excluded. Consequently, a possible increase in the mortality caused by malfunctioning of the referral system in after hours could go undetected. For the same reason, the choice of the variable used to classify patients (time of injury, time of arrival to 1st hospital or time of arrival to definitive hospital) could also influence the results.

Finally, the detailed definition of the working time should not be fixed but variable. It should depend on the characteristics of the setting being analyzed. For instance, the resources available on a Saturday morning may resemble those of business time in some hospitals/systems and those of aftertime in others. Thus, the QI should be adapted accordingly.

The feasibility of an indicator is an important aspect. This is because 'measures based on data that are difficult to obtain must be extremely valuable or they will result in misspent resources' [30]. For this reason, trauma mortality inside and outside working hours appears feasible, as the necessary data are already part of the core set recommended by the Utstein Template (30-day mortality, time of 1^st ^emergency call or time of hospital arrival, and predictive model variables) [31,32].

As mentioned previously, the literature investigating the 'off-hour' effect is inconsistent and divided more or less equally between the positive and negative findings. Curiously enough, all the studies focusing on trauma yielded negative results (no difference). However, the opposite occurred for studies focusing on myocardial infarction, which is surprising as both these conditions share many features: time-dependency, early mortality and the importance of early and centralized care. A majority of the studies on trauma were conducted in Level 1 Trauma Centers. These studies used the time of arrival at the hospital to classify the patients and excluded patients transferred between hospitals. This could have lowered the chances of finding a difference, as elucidated above. The other explanation is that the quality of trauma care in those studies was just good enough to protect from the 'weekend effect'. This appears reasonable given that Level 1 Trauma Centers have immediate access to a full trauma team at all times, while interventional cardiologists are rarely in-house during off-hours.

## Conclusions

The evaluation of the 'off-hour' effect is a possible quality indicator for trauma care, which has interesting theoretical characteristics. The above-mentioned QI would not require validation against the outcome, as it is an outcome indicator. At the same time it could also provide information about the aspects of care that require improvement, in the manner of a process indicator. The diffusion of this QI will help to define its value more precisely. This is because the literature till date demonstrates that either the developed trauma systems are safe from the 'off-hour' effect or the way to assess them needs to be refined.

## Competing interests

The author declares that they have no competing interests.

## References

[B1] Stelfox HT Bobranska-Artiuch B Nathens A Straus SE Quality Indicators for Evaluating Trauma Care Arch Surg 2010 145 286 295 10.1001/archsurg.2009.289 20231631

[B2] Carr BG Reilly PM Schwab CW Branas CC Geiger J Wiebe DJ Weekend and Night Outcomes in a Statewide Trauma System Arch Surg 2011 in press 10.1001/archsurg.2011.60 21422328

[B3] Bell CM Redelmeier DA Mortality among patients admitted to hospitals on weekends as compared with weekdays N Engl J Med 2001 345 663 668 10.1056/NEJMsa003376 11547721

[B4] Barnett MJ Kaboli PJ Sirio CA Rosenthal GE Day of the week of intensive care admission and patient outcomes: a multisite regional evaluation Med Care 2002 40 530 9 10.1097/00005650-200206000-00010 12021679

[B5] Kostis WJ Demissie K Marcella SW Shao YH Wilson AC Moreyra AE Myocardial Infarction Data Acquisition System (MIDAS 10) Study Group Weekend versus weekday admission and mortality from myocardial infarction N Engl J Med 2007 356 1099 109 10.1056/NEJMoa063355 17360988

[B6] Peberdy MA Ornato JP Larkin GL Survival from in-hospital cardiac arrest during nights and weekends JAMA 2008 299 785 792 10.1001/jama.299.7.785 18285590

[B7] Kuijsten HA Brinkman S Meynaar IA Spronk PE van der Spoel JI Bosman RJ de Keizer NF Abu-Hanna A de Lange DW Hospital mortality is associated with ICU admission time Intensive Care Med 2010 36 1765 71 10.1007/s00134-010-1918-1 20549184 PMC2940016

[B8] Fang J Saposnik G Silver FL Kapral MK Investigators of the Registry of the Canadian Stroke Network Association between weekend hospital presentation and stroke fatality Neurology 2010 75 1589 96 10.1212/WNL.0b013e3181fb84bc 21041782

[B9] Laupland KB Ball CG Kirkpatrick AW Hospital mortality among major trauma victims admitted on weekends and evenings: a cohort study Journal of Trauma Management & Outcomes 2009 3 8 10.1186/1752-2897-3-8 19635157 PMC2731032

[B10] Carmody IC Romero J Velmahos GC Day for night: should we staff a trauma center like a nightclub? Am Surg 2002 68 1048 51 12516806

[B11] Carr BG Jenkins P Branas CC Wiebe DJ Kim P Schwab CW Reilly PM Does the trauma system protect against the weekend effect? J Trauma 2010 69 1042 7 10.1097/TA.0b013e3181f6f958 21068609

[B12] Crowley RW Yeoh HK Stukenborg GJ Ionescu AA Kassell NF Dumont AS Influence of weekend versus weekday hospital admission on mortality following subarachnoid hemorrhage. Clinical article J Neurosurg 2009 111 60 6 10.3171/2008.11.JNS081038 19231928

[B13] Arbabi S Jurkovich GJ Wahl WL Kim HM Maier RV Effect of Patient Load on Trauma Outcomes in a Level I Trauma Center J Trauma 2005 59 815 8 10.1097/01.ta.0000188390.80199.37 16374267

[B14] Guly HR Leighton G Woodford M Bouamra O Lecky F Trauma Audit and Research Network The effect of working hours on outcome from major trauma Emerg Med J 2006 23 276 80 10.1136/emj.2005.028746 16549573 PMC2579501

[B15] Clarke MS Wills RA Bowman RV Zimmerman PV Fong KM Coory MD Yang IA Exploratory study of the 'weekend effect' for acute medical admissions to public hospitals in Queensland, Australia Intern Med J 2010 40 777 83 10.1111/j.1445-5994.2009.02067.x 19811554

[B16] Lohr KN Donaldson MS Harris-Wehling J Medicare: a strategy for quality assurance, V. Quality of care in a changing health care environment QRB Qual Rev Bull 1992 18 120 6 1630793 10.1016/s0097-5990(16)30518-8

[B17] Donabedian A The Definition of Quality and Approaches to Its Assessment 1980 Ann Arbor, MI, Health Administration press

[B18] Lilford RJ Brown CA Nicholl J Use of process measures to monitor the quality of clinical practice BMJ 2007 335 648 50 10.1136/bmj.39317.641296.AD 17901516 PMC1995522

[B19] Matz R Outcomes Remain The Gold Standard. BMJ rapid response, Published 3 October 2007. Ref. Liliford RJ, Brown CA, Nicholl J. Use of process measures to monitor the quality of clinical practice BMJ 2007 335 648 650 10.1136/bmj.39317.641296.AD 17901516 PMC1995522

[B20] The Joint Commission - Find a health care organisation http://www.qualitycheck.org

[B21] Hospital Compare http://www.hospitalcompare.hhs.gov/

[B22] O'Reilly D El Turabi A Coats T Willett K Trauma research: An opportunity and a challenge Injury, Int J Care Injured 2011 42 117 118

[B23] Willis CD Stoelwinder JU Cameron PA Interpreting process indicators in trauma care: construct validity versus confounding by indication Int J Qual Health Care 2008 20 331 338 18603538 10.1093/intqhc/mzn027

[B24] Di Bartolomeo S Valent F Sanson G Nardi G Gambale G Barbone F Are the ACSCOT filters associated with outcome? Examining morbidity and mortality in a European setting Injury 2008 39 1001 1006 10.1016/j.injury.2008.04.009 18657809

[B25] Stelfox HT Straus SE Nathens A Bobranska-Artiuch B Evidence for quality indicators to evaluate adult trauma care: A systematic review Crit Care Med 2011 in press 10.1097/CCM.0b013e31820a859a 21317653

[B26] Lecky F Bryden D Little R Emergency intubation for acutely ill and injured patients Cochrane Database Syst Rev 2009 10.1002/14651858.CD001429.pub2 PMC7045728 18425873

[B27] Maclure M The case-crossover design: a method for studying transient effects on the risk of acute events Am J Epidemiol 1991 133 144 153 1985444 10.1093/oxfordjournals.aje.a115853

[B28] Peden M The world report on road traffic injury prevention 2004 Geneva: World Health Organization

[B29] Lossius HM Kristiansen T Ringdal KG Rehn M Inter-hospital transfer: the crux of the trauma system, a curse for trauma registries Scandinavian Journal of Trauma, Resuscitation and Emergency Medicine 2010 18 15 10.1186/1757-7241-18-15 20233410 PMC2847963

[B30] Dimick JB What makes a "good" quality indicator? Arch Surg 2010 145 295 10.1001/archsurg.2009.291 20329352

[B31] Ringdal KG Coats TJ Lefering R Di Bartolomeo S Steen PA Røise O Handolin L Lossius HM The Utstein Template for Uniform Reporting of Data following Major Trauma. A joint revision by SCANTEM, TARN, DGU-TR, and RITG Scand J Trauma Resusc Emerg Med 2008 16 7 10.1186/1757-7241-16-7 18957069 PMC2568949

[B32] Brohi K The Utstein template for uniform reporting of data following major trauma: A valuable tool for establishing a pan-European dataset Scand J Trauma Resusc Emerg Med 2008 16 8 10.1186/1757-7241-16-8 18957070 PMC2568947

